# Utilization of Complementary and Alternative Medicine among Korean Elite Athletes: Current Status and Future Implications

**DOI:** 10.1155/2021/5572325

**Published:** 2021-02-22

**Authors:** Bo-Young Youn, Seongwan Ju, Shinhyoung Joo, Hoseok Kang, Kiyoung Jeon, Chunhoo Cheon, Ho-Yeon Go, Seong-Gyu Ko

**Affiliations:** ^1^Department of Global Public Health and Korean Medicine Management, Graduate School, Kyung Hee University, Seoul 02447, Republic of Korea; ^2^Department of Korean Medicine, Graduate School, Kyung Hee University, Seoul 02447, Republic of Korea; ^3^Department of Physical Education, College of Education, Dongguk University, Seoul 04620, Republic of Korea; ^4^Department of Judo Instructor Education, College of Martial Arts, Yong In University, Yongin 17092, Republic of Korea; ^5^Department of Preventive Medicine, College of Korean Medicine, Kyung Hee University, Seoul 02447, Republic of Korea; ^6^Department of Internal Medicine, College of Korean Medicine, Semyung University, Chungju 27429, Republic of Korea

## Abstract

The objective of the study was to explore the status of usage of complementary and alternative medicine (CAM) among Korean elite athletes. A survey was emailed to all Korean national sports federations recognized by the International Olympic Committee and the Olympic Council of Asia. A total of 705 Korean elite athletes participated in this study. The athletes had to be any of the following to participate in this survey: elite intercollegiate athletes, professional athletes, and national team athletes. 83.3% of the participants stated that they have previously experienced Korean medicine (KM). Compared to the general population in Korea, athletes had more experience (general population = 73.8%). The participants without experience mentioned that they either did not need any KM treatments (39.8%) or lacked information (39.8%) regarding KM treatments. The primary reason for the utilization of KM was the effectiveness of treatments. Therefore, 70.8% of the participants have mentioned recommending KM to others. Generally, athletes are worrisome that the consumption of herbal medicine may not be doping-free; however, it is vital to note that 62% of the participants expressed that prescribed herbal medicine is safe. Overall, this research demonstrates a high prevalence of KM usage by intercollegiate, professional, and national team athletes in Korea. Hence, this study's results may serve as the foundation in future research directions for promoting KM among Korean elite athletes.

## 1. Introduction

The use of complementary and alternative medicine (CAM) is continuously growing worldwide. The global CAM market was valued at 55.2 billion dollars in 2018, and it is expected to reach 163.3 billion dollars by 2025, at a Compound Annual Growth Rate (CAGR) of 16.8% between 2019 and 2025 [1].

Korean medicine (KM), Korea's own traditional medicine, has been building trust for over a decade. The popularity of KM tremendously increased from 45.8% to 69.3% among the general population between 2008 and 2011 [2], and the average number of outpatients per person for KM increased 63% from 0.81 in 2008 to 1.32 in 2013 [3]. Furthermore, the 2017 Korean Medicine Utilization and Herbal Medicine Consumption Survey, which is the most recent survey, reported 73.8% of the respondents had used KM [4].

Interest and use of complementary and alternative medicine treatments have also been growing in sports medicine. Pike mentioned that 60% of females and 10% of males had sought to use CAM therapies among the 200 amateur rowers for injury risk, pain management, and competing while injured [5]; Nichols and Harrigan found that 56% of the 309 Division 1 university athletes in the National Collegiate Athletic Association had reported receiving CAM therapies [6]; Lee et al. indicated that treating and preventing sports injuries for the Korean national volleyball team was effective with various KM modalities [7]. Additionally, both professional and recreational athletes informed that the therapies improve or assist athletic performance [8].

Although the prevalence of complementary and alternative medicine has increased for athletes in most countries, a detailed investigation of the factors of elite athletes' usage has yet to be widely studied. Hence, the study aimed to analyze the current utilization of KM among Korean elite athletes and find valuable data for future studies.

## 2. Materials and Methods

### 2.1. Study Design

An online questionnaire was created using Google forms survey. The survey web-link was emailed to all Korean national sports federations recognized by the International Olympic Committee and the Olympic Council of Asia. Then, the federations distributed the weblink to athletes. The research period was from September 7 to October 15, 2020.

### 2.2. Ethical Considerations

All participants had to acknowledge by clicking a box within the Google survey form that the data would only be used for research purposes only that all responses were confidential. This study involved no more than minimal risk to human subjects. For this reason, this study was exempt from the Institution Review Board (IRB) review by the IRB of the Kyung Hee University (No. KHSIRB-20-299-EA). In addition, the athletes volunteered to participate in this survey among the recruited athletes. The recruited athletes had an option not to participate without any penalties or harm.

### 2.3. Participants

A total of 705 elite athletes participated in this study. The athletes had to be any of the following to participate in this survey: elite intercollegiate athletes, professional athletes, and national team athletes.

### 2.4. Measures

The questionnaire was designed based on a combination of the 2017 Korean medicine utilization and herbal medicine consumption survey, and in-depth consultations from various national team coaches, trainers, retired elite athletes, and various sports associations. The Korean Medicine Utilization and Herbal Medicine Consumption Survey has been conducted since 2008 by the Ministry of Health and Welfare of South Korea. The total number of participants for the 2017 survey was 5,000; the general population data was retrieved from the survey report to compare results obtained from the athletes [9].

#### 2.4.1. Experience of KM

The questions for the experience of KM during an entire career as elite athletes were organized as follows: have you ever experienced KM before, what are the reasons for having no experience for KM, how familiar are you with KM, how frequently do you use KM, which institution have you visited for KM, and how satisfied are you with overall KM experience. The second question was only asked to those who answered “no” to the previous experience of the KM question. If the participants answered “yes” to the first question, the rest of the four questions were followed.

#### 2.4.2. Reasons for Utilization of KM

The reasons for the utilization of the KM part of the survey asked three questions to the participants. The first question was about the reasons for using KM treatments for medical care; the participants were allowed to choose multiple answers among the choices: disc problem, sprains, back pain, frozen shoulder, arthritis, and improvement of health status. The second question was related to any of the formerly used KM modalities; the participants were allowed to choose multiple answers amid the six items: acupuncture, moxibustion, cupping, pharmacopuncture, Chuna manual therapy, and herbal medicine. The last question asked the participants for the primary reason for using KM. The participants were able to select one item among the following: treatments are effective, no burden on surgery or examination, fewer side-effects, the cost is low, close distance, and unique treatments.

#### 2.4.3. Recommendation of KM

The last part of the survey dealt with the recommendation of KM. There were two primary questions to ask: the future recommendation of KM to other athletes and the primary reason for the recommendation. The participants had to choose either yes or no for the first question. Only one choice was allowed for the second question among the following options: treatments are effective, no burden on surgery or examination, fewer side-effects, the cost is low, and close distance. The participants who chose a positive response were asked to select the significant reason for willingness to recommend to other athletes from the five items.

### 2.5. Statistical Analysis

Descriptive analysis was performed, and the chi-square test was also conducted to find any differences between the two groups. Multiple logistic regression analysis was used to examine categorical independent variables with one dichotomous dependent variable. The statistical analysis was done using Microsoft Excel and the *R* software (ver. 4.0.3, http://r-project.org/).

## 3. Results

### 3.1. General Characteristics of the Data

All 705 participants' data were analyzed for this study. Unfortunately, we are not able to provide an estimate of the response rate as it was not feasible to count how many sports associations and coaches forwarded the questionnaire to athletes. Based on the 2019 elite sports registration statistics-sports support portal from the Korea Sport and Olympic Committee, the total number of targeted athletes was 35,486 [10].

The 20 to 25 age group had the highest number of total participants (*n* = 410, 58.2%). The male athletes accounted for 56.3% (*n* = 397), and the female athletes accounted for 43.7% (*n* = 308) of the total. Amid the participants, 38.9% (*n* = 274) had careers between 11 and 15 years, and 34.6% (*n* = 244) had careers between 6 and 10 years. The details of the general characteristics are shown in Table 1. The athletes from 31 sports represented this study (Table 2). Tae Kwon-do athletes represented the highest (*n* = 106, 15%), and Judo athletes represented the second-highest (*n* = 84, 11.9%).

### 3.2. Experience of Korean Medicine Treatments

Overall, 83.3% of the participants stated that they have previously experienced Korean medicine. Compared to Korea's general population; athletes had more experience (general population = 73.8%; Table 3). The general population without KM experiences has expressed KM as non-essential (71.2%). Of the participating athletes, 39.8% have indicated KM treatments were unneeded, and 39.8% have said a lack of KM information was the main reason for not having any KM experiences. It is noteworthy that most athletes utilize KM treatments (one to two times per month; 39.3%) much more than the general population (less than two times per year). The Korean medicine clinics were chosen as the primary institution for both athletes and the general population. Among the participants, 64.9% were satisfied with KM treatments, which was slightly lower than the general population (76.2% satisfied).

### 3.3. Reasons for Utilization of Korean Medicine

The participants indicated that they had used KM treatments for various sprains (63%); the next most reason for utilization was to treat their back pain (52.6%; Table 4). According to both participants and the general population, they have chosen acupuncture therapy as the most frequent treatment method (95.8% vs. 90.2%, respectively). The second most frequent method of treatment was the same for both groups, choosing the cupping therapy. The least interested KM method was herbal medicine treatments (21.6%) for the athletes, while Chuna manual therapy was selected within the general population, accounting for 7.8%.

Additionally, both groups have mentioned that the primary reason for using KM was the positive effects of treatments (athletes = 59.5%; general population = 66.3%). The second-highest reason was no burden on having surgery or excessive examination (13.9%) for the athletes. Fewer side effects (16.4%) were chosen as the second-highest reason for the general population.

### 3.4. Recommendation of Korean Medicine

While the majority of athletes (70.8%) and the general population (61.7%) have both expressed to recommend KM treatments to others, the athletes have a higher inclination to recommend as the result showed a higher percentage for athletes. Moreover, both groups chose the positive effects of KM treatments as the primary reason to recommend (athletes = 69.2%; general population = 55.4%, Table 5). The athletes indicated no burden on having surgery or excessive examination (13.6%) as the second-highest; the general population chose fewer side effects (11.3%) as the second-highest.

### 3.5. Issues between the Consumption of Herbal Medicine and Doping

Generally, athletes are worrisome that herbal medicine consumption may not be doping-free; however, it is vital to note that 62% of the participants expressed that prescribed herbal medicine from KM clinics, KM hospitals, and pharmacies is safe. Even though the participants said that herbal medicine is safe from doping tests, athletes were still concerned about herbal medicine not being doping-free. Of the athletes who answered that herbal medicine is not safe, 35.3% mentioned that athletes' worrisome of the prescribed herbal medicine could not be doping-free. The others noted that the high cost of herbal medicine (27.2%), the uncomfortable method of herbal medicine consumption (16.2%), the bitter taste of herbal medicine (11.5%), and the consumption of other medication (9.8%) were the reasons for not seeking herbal medicine at the moment (Table 6).

### 3.6. Multiple Logistics Regression Analysis

The purpose of the multiple logistics regression analysis was to determine the influence factors of the utilization of KM within athletes' characteristics. The chosen categories are age, sex, health status, type of sports, and perception of KM supplements being doping-free. If any of the categories is greater than 1, it implies a higher association for using KM. Amid the chosen variables, age, sex, and health status were selected as the significant variables to find the health effects of using KM. The three variables have been commonly used as key determinants in numerous health utilization studies [11–13]. The type of sports and perception of KM supplements being doping-free were chosen as athletes are sensitive to taking any health supplements for doping issues.

Female athletes were more likely to have received KM, as compared with male athletes (3.53, 95% CI 2.18 to 5.72), and the 31–35 age group was more likely to have received KM than other age groups (3.3, 95% CI, 1.49–7.30). Athletes from the team sports were likely to have received KM compared to athletes from the individual sports (2.34, 95% CI 1.24 to 4.41). Athletes, who believed the KM herbal treatments or supplements were doping-free, were more likely to use KM than those who did not believe (1.38, 95% CI 0.9 to 2.13). Moreover, athletes in bad health conditions were more likely to use KM than others (2.72, 95% CI 1.19 to 6.23; Table 7).

## 4. Discussion

The present study was proposed to explore Korean medicine's current state of utilization among elite intercollegiate, professional, and national team athletes. The questionnaire was designed similar to the 2017 Korean Medicine Utilization and Herbal Medicine Consumption Survey in order to compare both results to discover crucial dissimilarities. The study found that athletes had a higher percentage of KM utilization than the general population and had visited more than the general population, which parallels previous literature that athletes, as the highest users of CAM, may help pioneer population trends in CAM use [14].

Regarding the experience of KM, female athletes used it more than the male athletes in this study. Compared to the results, the general population had the same findings; female participants used more than male participants. The result was the same as the norm as women are generally more favorable toward seeking and experience CAM than men in most countries [15].

The 71.2% of the general population indicated that KM is unneeded for not having any previous experiences. On the other hand, athletes equally selected (both 39.8%) “not being able to gain information” and “not finding the need” to use KM. On the report of athlete's handling of medical knowledge research, it is known that physicians or physiotherapists are the most trusted sources of medical information; therefore, athletes may not have the opportunities to comprehend how KM or CAM therapies and treatments work, considering physicians or physiotherapists provide knowledge focuses mainly on medical standards [16].

The popularity of complementary and alternative medicine has been rising in recent years as specific CAM therapies are effective and accepted into mainstream practice [17]. This study confirms the previous statement since more than half of the study participants have expressed the main reason for using KM modalities for their positive effects. The athletes have predominantly used KM modalities for treating musculoskeletal injuries rather than preventing or enhancing conditions from the study results for reasons of using KM.

Acupuncture was chosen as the highest utilized treatment in this study; it is highly effective for treating musculoskeletal conditions. Previous studies demonstrated that acupuncture is a positive method for treating various sports-related musculoskeletal injuries [18]. For instance, acupuncture was applied to treat Taiwanese wrestlers' injuries, and KM national volleyball team doctors used acupuncture the most to treat injuries [19, 20]. It was unique to find that athletes (27.8%) had more experience of the Chuna manual therapy than the general population (7.8%). Chuna manual therapy is a manipulation treatment that corrects the displacement of the structures while balancing the whole body; the aforementioned volleyball team doctors have used the Chuna manual therapy as the second most treatment [21].

Athletes' recommendation of KM may have a few vital reasons. First, athletes indicated that the treatments are effective as athletes are inclined to “try all possibilities to get back on the field” [22]. The second reason may be the strong inclination to maintain injuries rather than having surgery, as the athletes have mentioned that using KM is associated with no burdens for surgery. According to Kraemer et al., athletes are aware that the severity of an injury and the surgical care complexity affect the recovery rate [23]. That being said, long-term recovery may have a negative impact on athletes to remain competitive if the athletes may not have the same strength before any medical or surgical care.

The study found that 62% of the participants have said that the KM herbal supplements are doping-free. This result was surprising as a vast number of athletes would think the herbal supplements are not doping-free. Additionally, 35.3% of the athletes have answered that the supplements may not be doping-free when the reasons for not being in favor of KM herbal treatments or supplements were asked. The second-highest reason was the high cost of herbal treatments or supplements (27.2%). Although the logistic regression analysis specified that athletes who thought KM herbal treatments or supplements were doping-free were not statistically significant, the outcome may have been different if the dependent variable was merely focused on herbal medicine consumption. There are several reasons why athletes are inclined to hinder taking herbal supplements. First, the lack of knowledge could be the main reason since the greater the knowledge, the more the athletes are willing to take supplements [24]. Second, there are not enough educational programs available in every country [25]. Lastly, coaches may not have comprehensive knowledge to give athletes appropriate recommendations [26].

A limitation of the study is that certain sports were better represented, having an uneven number of participants and athletes' distribution, than others even though the study included a wide range of participants from various sports categories. As taking any record of athletes' privacy information was allowed, there might be a possibility for multiple responses from the same person. However, it can be assumed the low probability of athletes completing the survey more than once since any incentives or rewards were not provided to participants.

Dealing with such a large data along with the numerous variables, it was impossible to explain each athlete's characteristics toward using KM treatments. As coaches and athletes are continually seeking new methods to improve performance, it may be meaningful to research if any KM treatments help boost athletic performance for future studies. Furthermore, additional investigation is needed to explore the effects of using the KM treatments throughout athletes' careers. Further, not being able to include the differences in Western medicine utilization between athletes and the general population may be the weakness point.

Based on the results of this study, there are three implications to promote KM to athletes. Although athletes are aware of its positive effects, KM treatments have been heavily focused on treating injuries. As there are numerous KM modalities to enhance athletic performance, KM physicians need to inform and provide various options to pursue. Second, KM physicians may treat athletes the same as the general population; perhaps, KM physicians should propose special treatment options for athletes across the care continuum. Lastly, the results show that athletes who have KM experience are likely to consider herbal treatments or supplements. Utilization of KM herbal medicine can be substantially beneficial, from preventing injuries to enhancing athletic performance; more importantly, KM physicians have been researching vigorously to find the evidence for KM herbal medicine for athletes to use safely during the course of the career [27]. Hence, vibrant promotion campaigns of KM from the coaches, athletic trainers, and team doctors to athletes will not only increase the usage of KM but also increase more appropriate treatment, trust, and perception of herbal treatments and supplements in the Korean sports community.

## 5. Conclusion

This research demonstrates a high prevalence of KM usage by intercollegiate, professional, and national team athletes in Korea. Additionally, athletes have a higher prevalence of utilization than the general population. Most Korean elite athletes have experienced KM; the frequently used treatment types are acupuncture, cupping, moxibustion, pharmacopuncture, chuna manual therapy, and herbal medicine treatments. The elite athletes have shown optimistic attitudes in taking herbal medicine, yet some athletes still believe that herbal supplements are not doping-free. Overall, elite athletes are satisfied with KM treatments and are willing to recommend it to other athletes who have never experienced KM. This study might help understand the current prevalence of CAM usage in sports medicine. This study's results may serve as the foundation in future research directions for promoting KM among Korean elite athletes.

## Figures and Tables

**Table 1:** General characteristics of the data.

Characteristics	Frequency (n)	Percentage
Total	705	100
Gender		
Male	397	56.3
Female	308	43.7
Age group (years)		
20–25	410	58.2
26–30	190	27
31–35	92	13
Above 35	13	1.8
Career (years)		
1–5	38	5.4
6–10	244	34.6
11–15	274	38.9
16–20	25	3.5
21–25	19	2.7
Above 25	12	1.7
Disability status		
At least one disability	8	1.1
No disability	697	98.9

**Table 2:** Sports categories of the participants.

Categories	Frequency (n)	Percentage
Archery	4	0.6
Badminton	10	1.4
Baseball	22	3.1
Basketball	31	4.4
Bowling	21	3.0
Boxing	1	0.1
Canoe	4	0.6
Cycling	9	1.3
Fencing	12	1.7
Golf	19	2.7
Gymnastics	19	2.7
Handball	4	0.6
Ice hockey	13	1.8
Inline skating	4	0.6
Judo	84	11.9
Parkour	2	0.3
Rowing	13	1.8
Sailing	28	4.0
Shooting sports	53	7.5
Short-track skating	7	1.0
Soccer	53	7.5
Speed skating	9	1.3
Squash	16	2.3
Swimming	15	2.1
Table Tennis	12	1.7
Tae kwon-do	106	15.0
Tennis	5	0.7
Track and field	29	4.1
Volleyball	9	1.3
Weightlifting	15	2.1
Wrestling	76	10.8

**Table 3:** Experience of Korean medicine treatments.

Questions	Answer choice	Elite athletes	General population	*P* value
Q. Have you experienced KM previously?	Yes	83.3	73.8	0.143
	No	16.7	26.2	
Q. What are the reasons for having no experience for KM?	No info for KM	39.8	9.5	<0.001
	Treatment is expensive	5.5	4.2	
	Worrisome for safety of herbal medicine	7.0	3.4	
	KM treatments are too much	7.8	11.6	
	No need for KM	39.8	71.2	
Q. How familiar are you with KM?	Extremely familiar	17.3	4.5	<0.001
	Very familiar	9.8	30.4	
	Moderately familiar	38.7	28.3	
	Slightly familiar	25.5	34.7	
	Not at all familiar	8.7	2.2	
Q. How frequently do you use KM?	3 times per week	2.0	1.0	<0.001
	1–2 times per week	8.3	4.2	
	1–2 times per month	39.3	10.0	
	3–4 times per year	29.5	21.1	
	Less than two times per year	21.0	63.7	
Q. Which institution have you visited for KM?	KM clinic	96.9	96.9	1
	Pharmacy	11.5	8.3	0.619
	Public health center	2.8	4.7	0.738
	Herbal medicine pharmacy store	11.9	10.2	0.894
	KM hospital	22.6	15.5	0.303
Q. How satisfied are you with overall KM experience?	Very satisfied	32.4	8.7	<0.001
	Satisfied	32.5	67.5	
	Neutral	31.7	21.7	
	Dissatisfied	2.9	1.6	
	Very dissatisfied	0.5	0.4	

**Table 4:** Reasons for utilization of Korean medicine treatments.

Questions	Answer choice	Elite athletes	General population	*P* value
Q. What are the reasons for KM treatments?	Disc problem	17.9	12.7	0.409
	Sprains	63.0	37.3	<0.001
	Back pain	52.6	52.7	1
	Frozen shoulder	17.2	20.9	0.627
	Arthritis	25.3	20.7	0.545
	Improvement of health status	11.4	16.5	0.403
Q. Which KM treatments have you used?	Acupuncture	95.8	90.2	0.202
	Moxibustion	70.1	49.1	0.004
	Cupping	80.5	53.0	<0.001
	Pharmacopuncture	47.9	22.6	<0.001
	Chuna manual therapy	27.8	7.8	<0.001
	Herbal medicine	21.6	39.2	0.011
Q. What is the primary reason for using KM?	Treatments are effective	59.5	66.3	0.002
	No burden on surgery or examination	13.9	6.6	
	Less side effects	4.1	16.4	
	Cost is low	4.4	1.0	
	Close distance	9.8	1.6	
	Special treatments	8.3	8.0	

**Table 5:** Future recommendation of Korean medicine to others.

Questions	Answer choice	Elite athletes (%)	General population (%)	*P* value
Q. Are you willing to recommend KM to others?	Yes	70.8	61.7	0.226
	No	29.2	38.3	
Q. What is the primary reason to recommend to others?	Treatments are effective	69.2	55.4	0.002
	No burden on surgery or examination	13.6	11.3	
	Less side effects	6.5	27.9	
	Cost is low	4.2	2.1	
	Close distance	6.5	3.2	

**Table 6:** Athletes' opinions of utilization of herbal medicine from KM.

Questions	Answer choices	Percent
Q. Do you think that the prescribed herbal medicine from KM clinics, KM hospitals, and pharmacies is safe (doping-free)?	Yes	62
	No	38
Q. What is the biggest reason for not consuming herbal medicine or supplements?	Uncomfortable taking herbal medicine	16.2
	High cost of herbal medicine	27.2
	Worrisome of herbal medicine not being doping-free	35.3
	Bitter taste of herbal medicine	11.5
	Consumption of other medication	9.8

**Table 7:** Multiple logistic regression analysis of the chosen categories to evaluate the variables associated with experience of KM use in athletes.

Categories	Or (95% CI)
Sex	
Male	1
Female	3.53 (2.18–5.72)
Age	
20–25	1
26–30	1.49 (0.89–2.50)
31–35	3.3 (1.49–7.30)
>36	2.63 (0.56–12.42)
Type of sports	
Individual	1
Team	2.34 (1.24–4.41)
Perception of KM supplements being doping-free	
No	1
Yes	1.38 (0.9–2.13)
Health status	
Normal	1
Bad	2.72 (1.19–6.23)
Good	1.42 (0.91–2.21)

## Data Availability

The data are available upon request to the first author.

## References

[1] (2019). *Complementary and Alternative Medicine Market: By Intervention; by Distribution Channel: Global Industry Perspective, Comprehensive Analysis and Forecast, 2018-2025*.

[2] Kwon S., Heo S., Kim D., Jang S., Woo J.-M. (2017). Changes in trust and the use of Korean medicine in South Korea: a comparison of surveys in 2011 and 2014. *BMC Complementary and Alternative Medicine*.

[3] Kim D., Lim B. (2017). The trend of Korean medicine utilization in 2008-2013. *Journal of Society of Preventive Korean Medicine*.

[4] Ministry of Health and Welfare (2017). *Korean Medicine Utilization and Herbal Medicine Consumption Survey*.

[5] Pike E. C. J. (2005). Doctors just say “rest and take ibuprofen. *International Review for the Sociology of Sport*.

[6] Nichols A. W., Harrigan R. (2006). Complementary and alternative medicine usage by intercollegiate athletes. *Clinical Journal of Sport Medicine*.

[7] Lee Y.-S., Park D. S., Oh J. K., Kim S.-Y. (2020). Prediction model for utilization of complementary and alternative medicine for sports injuries among Korean elite collegiate athletes. *Integrative Medicine Research*.

[8] Evans L., Wadey R., Hanton S., Mitchell I. (2012). Stressors experienced by injured athletes. *Journal of Sports Sciences*.

[9] Ministry of Health and Welfare (2017). Korean medicine utilization and herbal medicine consumption survey report. https://www.koms.or.kr/page/research-result/reality-people.do?menu_no=14.

[10] Korea Sport and Olympic Committee Sports Portal (2019). *Status of Registrations Statistics for Elite Athletes*.

[11] Saini P., McIntyre J., Corcoran R. (2019). Predictors of emergency department and GP use among patients with mental health conditions: a public health survey. *British Journal of General Practice*.

[12] Baldani M. H., Antunes J. L. F. (2011). Inequalities in access and utilization of dental services: a cross-sectional study in an area covered by the family health strategy. *Cadernos de Saúde Pública*.

[13] Lee S., Grant D. (2009). The effect of question order on self-rated general health status in a multilingual survey context. *American Journal of Epidemiology*.

[14] Koh B., Freeman L., Zaslawski C. (2012). Alternative medicine and doping in sports. *Australasian Medical Journal*.

[15] Frass M., Strassl R. P., Friehs H., Müllner M., Kundi M., Kaye A. D. (2012). Use and acceptance of complementary and alternative medicine among the general population and medical personnel: a systematic review. *The Ochsner Journal*.

[16] Gerbing K.-K., Thiel A. (2016). Handling of medical knowledge in sport: athletes’ medical opinions, information seeking behaviours and knowledge sources. *European Journal of Sport Science*.

[17] Audette J. F., Bailey A. (2007). *“Complementary and Alternative Medicine and the Athlete,” Clinical Sports Medicine*.

[18] Malone M. A., Gloyer K. (2013). Complementary and alternative treatments in sports medicine. *Primary Care: Clinics in Office Practice*.

[19] Lin Z.-P., Chen Y.-H., Chia F., Wu H.-J., Lan L. W., Lin J.-G. (2011). Episodes of injuries and frequent usage of traditional Chinese medicine for taiwanese elite wrestling athletes. *The American Journal of Chinese Medicine*.

[20] Yang C., Lee E., Kwon O., Lee J.-H. (2016). Management of sport injuries with Korean medicine: a survey of Korean national volleyball team. *Evidence-Based Complementary and Alternative Medicine*.

[21] Lee N.-W., Kim G.-H., Heo I. (2017). Chuna (or Tuina) manual therapy for musculoskeletal disorders: a systematic review and meta-analysis of randomized controlled trials. *Evidence-Based Complementary and Alternative Medicine*.

[22] Ernst E., Willoughby M., Weihmayr T. H. (1995). Nine possible reasons for choosing complementary medicine. *Perfusion*.

[23] Kraemer W., Denegar C., Flanagan S. (2009). Recovery from injury in sport: considerations in the transition from medical care to performance care. *Sports Health: A Multidisciplinary Approach*.

[24] Nieper A. (2005). Nutritional supplement practices in UK junior national track and field athletes. *British Journal of Sports Medicine*.

[25] Jovanove P., Dordic V., Obradovic B. (2019). Prevalence, knowledge and attitudes towards using sports supplements among young athletes. *Journal of the International Society of Sports Nutrition*.

[26] Torres-McGehee T. M., Pritchett K. L., Zippel D., Minton D. M., Cellamare A., Sibilia M. (2012). Sports nutrition knowledge among collegiate athletes, coaches, athletic trainers, and strength and conditioning specialists. *Journal of Athletic Training*.

[27] Yun S. J., Je J. J., Lee H., Kim Y. J., Lee H. S. (2015). Anti-doping of herbal medicine in. 015. *Journal of Sports Korean Medicine*.

